# Effect of play between bracket and archwire on anterior tooth movement in sliding mechanics: A three-dimensional finite element study

**DOI:** 10.1177/1758736012461269

**Published:** 2012-10-17

**Authors:** Jun-ya Tominaga, Pao-Chang Chiang, Hiroya Ozaki, Motohiro Tanaka, Yoshiyuki Koga, Christoph Bourauel, Noriaki Yoshida

**Affiliations:** 1 Department of Orthodontics and Dentofacial Orthopedics, Nagasaki University Graduate School of Biomedical Sciences, Nagasaki, Japan; 2 School of Dentistry, Rheinische Friedrich-Wilhelms-University Bonn, Bonn, Germany

**Keywords:** Play, sliding mechanics, power arm, finite element method, deformation of archwire

## Abstract

**Objectives:**

The aim of this study was to clarify the effect of the play between the bracket and the archwire on anterior tooth movement subjected to the retraction force from various lengths of power arms in sliding mechanics.

**Materials and Methods:**

A three-dimensional finite element method was used to simulate en masse anterior tooth retraction in sliding mechanics. The displacements of the maxillary incisor and the archwire deformation were calculated when the retraction force was applied.

**Results:**

When a play did not exist, bodily movement was obtained at 5.0 mm length of power arm. In case a play existed, bodily movement was observed at the power arm length of 11.0 mm.

**Conclusions:**

In the actual clinical situation, a bracket/archwire play and the torsion of the archwire within the bracket slot should be taken into consideration to prescribe an optimal power arm length and to achieve effective anterior tooth movement.

## Introduction

In recent years, the demand for speedy, effective, and accurate orthodontic treatment system has been increasing. In such background, sliding mechanics in combination with implant anchorage has become more and more applicable all around the world.^1–8^ However, the mechanics of the system for achieving the desired tooth movement during space closure in sliding mechanics has not been established.

In addition to the combination system of sliding mechanics and implant anchorage, utilization of power arms attached onto an archwire allows controlled anterior tooth movement, which is essential to attain an individualized treatment plan. That is, the force system for the desired type of tooth movement, such as lingual crown tipping, bodily movement, or lingual root tipping can be easily carried out by attaching various lengths of power arm to the archwire in sliding mechanics.^9–13^

Although many studies have been carried out to investigate the various biomechanical factors affecting tooth movement in sliding mechanics, such as the flexural rigidity of the archwire, friction, and the height of retraction force,^10–15^ optimal loading conditions for controlled movement of anterior tooth by combined use of sliding mechanics and power arms have not been fully understood. Several calculations have been made by using the finite element method (FEM) on tooth displacement in a situation, in which single canine retraction or en masse retraction is performed in sliding mechanics. Friction between bracket and archwire was, however, simulated by using spring elements between tooth and archwire in several studies.^16–18^ Therefore, the mechanical condition is not sufficiently likely to approximate an actual clinical situation. To our knowledge, there is only one study that simulates en masse retraction under conditions where brackets were modeled and a coefficient of friction was given between brackets and an archwire.^19^ However, the simulation was not performed under the condition where play between the archwire and the brackets exists. In other words, a full-size archwire without any play was used in the study in Ref. 19. There has been no study in which a contact between a surface of a bracket slot and an archwire was taken into consideration due to difficulty in setting such a mechanical condition. The purpose of this study was to clarify the effect of the play between the bracket and the archwire on anterior tooth movement subjected to a retraction force and how power arms affect the archwire deformation and the controlled movement of anterior teeth in sliding mechanics by means of three-dimensional (3D) FEM.

## Materials and methods

### 3D FEM

**Figure 1.**
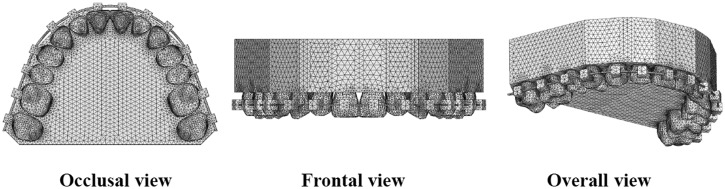
3D finite element model of maxillary dentition, including PDL, alveolar bone, brackets and archwire. The first premolar was taken out for the actual simulations.

Using a multi-image cone beam computed tomography (CT) scanner (3DX; J. Morita, Kyoto, Japan), CT images of upper 14 teeth were taken. The CT images were saved as digital imaging and communication in medicine (DICOM) data and exported to 3D image processing and editing software (Mimics 10.02; Materialize Software, Leuven, Belgium). The 3D solid model was created and converted to a 3D finite element (FE) model by using FE analysis pre- and postprocessor software (Patran 2008r1; MSC Software Corp., Los Angeles, CA, USA). Each 3D FE model for periodontal ligament (PDL), alveolar bone, bracket, archwire, and power arm was separately constructed using the same software. As performed in most FE studies with a set of multiple teeth, the PDL had a uniform thickness of 0.2 mm,^20–22^ although the alveolus is in the shape of an hourglass. The bracket height of maxillary central incisor was sited according to a prescription, which was 4.5 mm in height from the incisal edge of the tooth.^23^ Two power arms were attached onto the archwire bilaterally at the site between lateral incisor and canine. All the brackets were with 0.018 × 0.025-in slot, and 0.018 × 0.025-in and 0.016 × 0.022-in stainless steel archwires were modeled, respectively. Based on these 3D solid models, an FE mesh was created to make a node-to-node connection between tooth, PDL, and alveolar bone. An FE mesh of the archwire was created separately from the bracket to allow the archwire to slide through the bracket slots. The 3D FE model consisted of 414,900 ten-noded isoparametric tetrahedral solid elements and 81,459 nodes, or 428,004 elements and 84,861 nodes, respectively, according to whether play between the archwire and the brackets did exist or did not exist (Figure 1).

### Material parameters

The material parameters used in this study are represented in Table 1.^21,22^ In order to simplify the model and to reduce the time for analysis, the same properties were given to the archwire, power arm, and bracket. The structures of the tooth, alveolar bone, and PDL were modeled as being homogenous and isotropic for the same reason.

**Table 1.** Material parameters required within the finite element models^21,22^

Material	Young’s modulus (MPa)	Poisson’s ratio
Tooth	20,000	0.30
PDL	0.05	0.30
Alveolar bone	2000	0.30
Archwire/power arm/bracket	200,000	0.30

PDL: periodontal ligament.

### Experimental conditions

**Figure 2.**
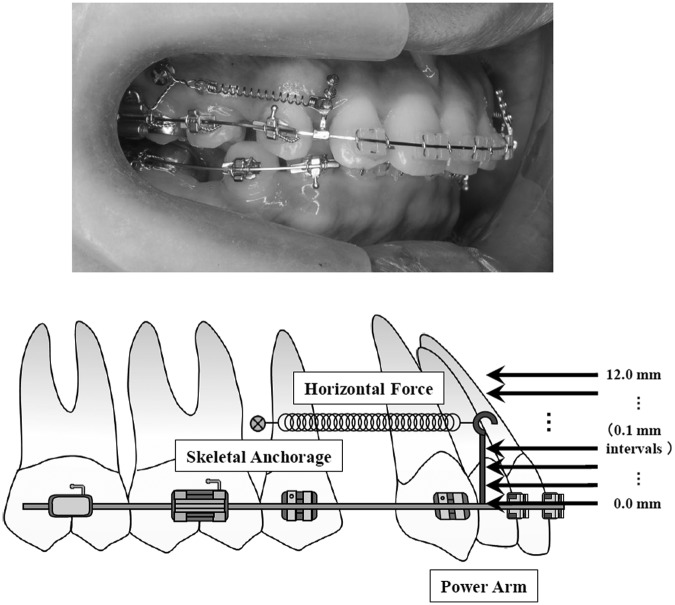
Intraoral picture and the illustration of the retraction of the anterior tooth using various lengths of power arm and implant anchorage in sliding mechanics

**Figure 3.**
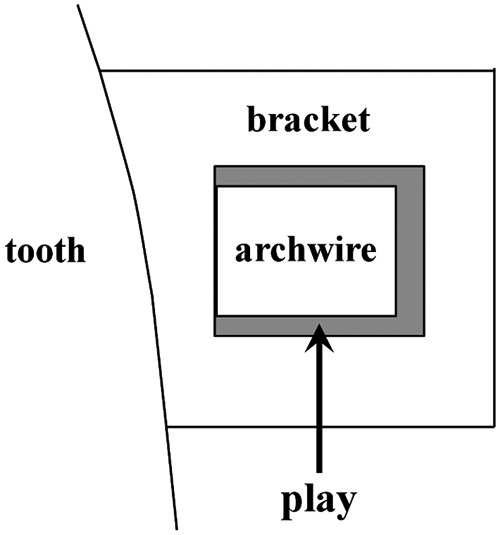
Illustration of the cross section of a play constructed in the model.

Assuming that the case model was diagnosed as maxillary protrusion and bilateral maxillary first premolar, extractions were indicated. That is, the model included 12 teeth, and two titanium miniscrew or miniplate implants, used as a skeletal anchorage, were inserted at both sides of the buccal region of the posterior teeth. The horizontal retraction force of 150*g* was applied from the implant anchorage to the power arms on both sides. Length of the power arms were changed from 0 to 12 mm with 0.1 mm intervals from the bracket slot level (Figure 2). The model was restrained in 6 degrees of freedom at the bottom of the alveolar bone to avoid sliding movement of the entire model. Coefficient of friction between a bracket slots and the archwire was assumed to be 0.2.^24–26^ A cross-sectional view indicating the boundary between bracket and archwire is illustrated in Figure 3. An archwire contacted the bottom surface of bracket slots, assuming that the archwire was fully engaged into the brackets. Under these conditions, 3D FE analysis was performed by using a 3D FE program (Marc; MSC Software Corp.). We investigated how the archwire was deformed and, consequently, how the maxillary central incisor moved, for the prediction of the maxillary central incisor movement is one of the most important factors for the orthodontist to diagnose and plan orthodontic treatment. Then, a comparison of the results obtained with 0.016 × 0.022-in and 0.018 × 0.025-in stainless steel archwire was performed.

## Results

**Figure 4.**
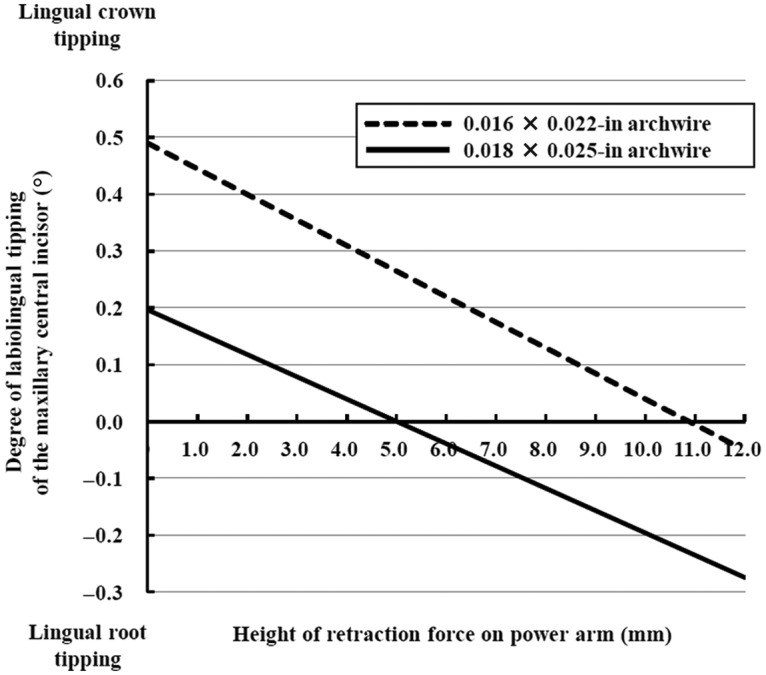
Degree of the central incisor’s rotation as a function of the height of retraction force on power arm. Positive signs indicate lingual crown tipping, whereas negative signs indicate lingual root tipping.

The relationship between the degree of labiolingual tipping of the maxillary central incisor and height of the retraction force on the power arm is shown in Figure 4.

In case the 0.018 × 0.025-in full-size stainless steel archwire was used, that is, no play between the bracket and the archwire existed, as shown by a solid line in Figure 4, lingual crown tipping of the maxillary central incisor was observed when the retraction force was set at 0 mm, which is the level of the bracket slot. The direction of tooth rotation changed from lingual crown tipping to lingual root tipping as the height level of the retraction force on the power arm was moved apically from the bracket slot level. At the height level of 5.0 mm, no rotation was produced and bodily movement occurred. Lingual root tipping of the incisor was observed when the retraction force was set above 5.0 mm.

When the 0.016 × 0.022-in stainless steel archwire was used, that is, a play existed, as shown by a dotted line in Figure 4, though the rotational tendency was the same as in the case previously mentioned, bodily movement of the incisor was achieved at the height level of the retraction force of around 11.0 mm.

**Figure 5.**
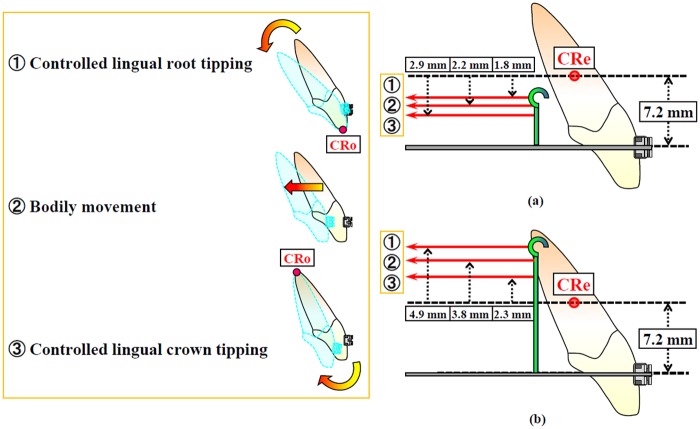
Loading conditions when controlled anterior tooth movements are performed (a) in case of using 0.018 × 0.025-in stainless steel archwire (without play); (b) in case of using 0.016 × 0.022-in archwire (with play). CRe, center of resistance; CRo, center of rotation.

Figure 5 shows the loading conditions when controlled anterior tooth movements, such as controlled lingual root tipping, bodily movement, and controlled lingual crown tipping, could be achieved. The controlled lingual root tipping is, by definition, the type of tooth movement in which the tooth tips around its incisal edge as the center of rotation (CRo). On the other hand, controlled lingual crown tipping indicates the movement, in which the tooth tips with its root apex as the CRo.

In case the 0.018 × 0.025-in archwire was used, controlled lingual root tipping was carried out at the height of the retraction force of 5.4 mm. At a level of 5.0 mm height, bodily movement was achieved. The controlled lingual crown tipping was observed at a height level of the retraction force of around 4.3 mm (Figure 5(a)).

When the 0.016 × 0.022-in archwire was used, the controlled lingual root tipping was obtained at 12.1 mm height. The bodily movement was produced at 11.0 mm height of retraction force. The controlled lingual crown tipping occurred at a level of 9.5 mm height (Figure 5(b)).

**Figure 6.**
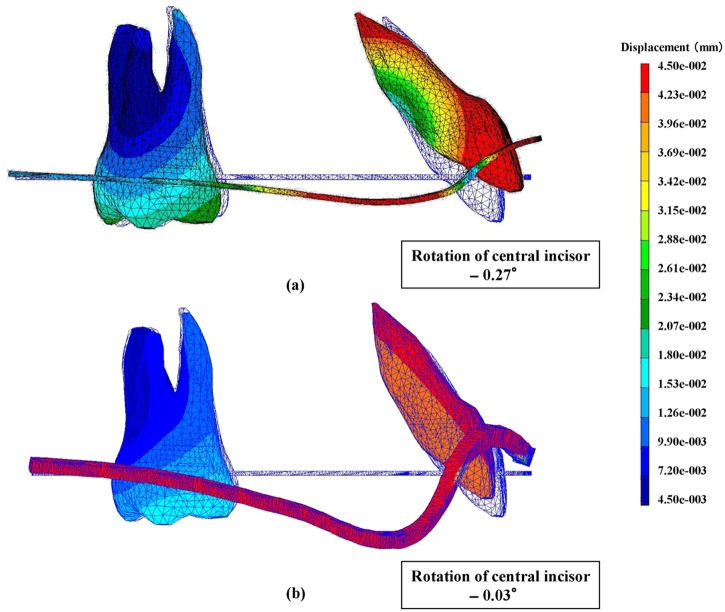
Displacement of the maxillary central incisor and the deformation of the archwire after the same height of retraction force (12.0 mm) was applied. For a better understanding of the displacement of the tooth and deformation of the archwire, these movements are magnified 50 times. Initial position of the tooth and the archwire are indicated by blue lines. (a) without play; (b) with play.

The deformation of the archwire and the resultant displacement of the maxillary central incisor following application of the retraction force from 12-mm power arm are shown when 0.018 × 0.025-in stainless steel archwire was used, that is, a play did not exist (Figure 6(a)), and when 0.016 × 0.022-in archwire was used, that is, a play existed (Figure 6(b)). For a better understanding of the deformation of the archwire and tooth displacement, these displacements were magnified 50 times, and the central incisor and the first molar were only described. Initial position of the tooth and the archwire is indicated by blue lines.

When a play did not exist, the anterior segment of the archwire was deformed upward and the root of the maxillary central incisor was substantially moved to the lingual and the crown to the labial (Figure 6(a)). The degree of labiolingual tipping was −0.27° (lingual root tipping).

When a play existed, the incisor showed less lingual root tipping, −0.03°, and the anterior segment of the archwire was subjected to a larger torsional deformation as compared with when a play did not exist (Figure 6(b)).

## Discussion

In this study, it was found that the existence of the play between the bracket and the archwire has a significant impact upon the anterior tooth movement when the retraction force was applied from the power arm in sliding mechanics.

Even if the same height of the horizontal force was applied, that is, the same length of power arm was used to retract anterior teeth, there were great discrepancies in tendency of labiolingual tipping of the incisor between the cases with 0.016 × 0.022-in and 0.018 × 0.025-in stainless steel archwires as shown in Figure 4. Although controlled movement of anterior teeth can be easily achieved from lingual crown to lingual root tipping with a full-size archwire that has no play, lingual root tipping can hardly be generated at any height of the power arm in clinical conditions when play existed.

From a biomechanical point of view, the relationship between the line of action of a force and the location of the center of resistance (CRe) of a tooth determines the type of tooth movement, such as lingual crown tipping, bodily movement, or lingual root tipping.^9,27,28^ However, as shown in Figure 5, tooth movements analyzed in this study were not in agreement with that concept based on biomechanical principles, wherein a single force passing through the CRe causes bodily tooth movement. Since the location of the CRe of the incisor was determined to be at the level of 7.2 mm apically from the bracket slot from FE analysis in the present study (Figure 5), bodily movement was expected to be produced at the height of 7.2 mm. Moreover, FE analysis showed that bodily movement of the incisor occurs at the height of 5.0 mm, which is 2.2 mm incisal to the level of CRe, with 0.018 × 0.025-in archwire (Figure 5(a)). On the other hand, bodily movement occurs at the level of 11.0 mm, which is 3.8 mm apical to the CRe, with 0.016 × 0.022-in archwire under the condition that a play exists (Figure 5(b)). The concept based upon the theoretical considerations does not work with the multibracket appliances.

When a 0.018 × 0.025-in stainless steel archwire is used, power arms shorter in length than the height of CRe are required to achieve any type of tooth movement, including controlled lingual root tipping, bodily movement, and controlled lingual crown tipping. On the contrary, power arms longer in length than the height of the CRe are required to achieve controlled movement of anterior tooth with a 0.016 × 0.022-in archwire. There were great discrepancies in types of anterior tooth movement between the cases with the two different sizes of archwires. This may be mainly due to the existence of play between the bracket and the archwire. Tominaga et al.^19^ reported that when relatively long power arms were used, a substantial amount of bending moment is generated at the base of the power arms as a cantilever effect. As a result, the anterior segment of the archwire was raised upward, causing lingual root tipping with a full-size 0.018 × 0.025-in stainless steel archwire.

**Figure 7.**
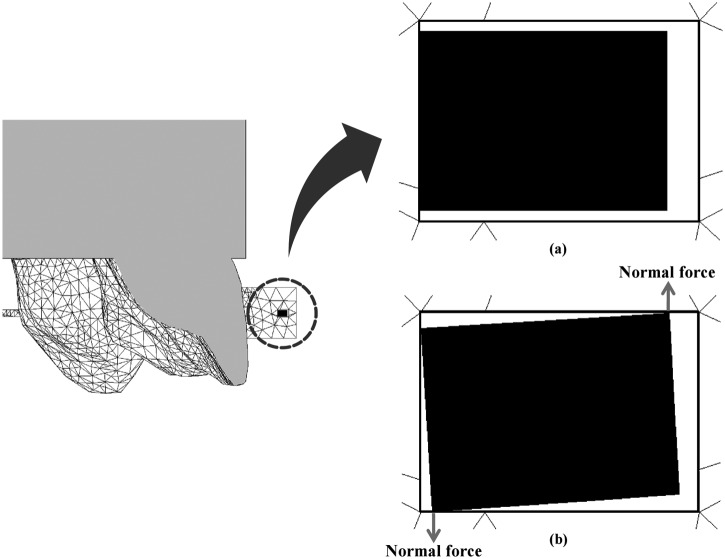
Sagittal cross section at the mesial surface of the maxillary central incisor bracket in the model which has a play. (a) before orthodontic force applied; (b) after the application of the force at the level of 12.0 mm.

Although the present study delivered similar results with the full-size archwire, when the 0.016 × 0.022-in archwire was used, lingual root tipping was not obviously observed even with longer power arms. Since the archwire deformation was considered to have a major impact on the tooth movement, displacements of archwire and the resultant tooth displacement were analyzed when using 0.016 × 0.022-in and 0.018 × 0.025-in stainless steel archwires (Figure 6). In case of the full-size archwire, the torsion of the anterior segment of the archwire may be entirely transmitted to the bracket on the incisor, and lingual root tipping can be effectively achieved with long power arms (Figure 6(a)). Contrary to this, the torsion of the archwire is less likely to be transmitted effectively to the incisor, thereby causing less lingual root tipping when using the archwire that has a play in the bracket slot (Figure 6(b)). In order to elucidate the mechanism of generation of lingual root torque, the cross-sectional view of the interface between the archwire and the bracket on the maxillary central incisor was constructed at the application of the retraction force from power arms of 12 mm (Figure 7). When the archwire is twisted due to a bending moment by means of power arms, the diagonally opposite corners of the archwire contact the surface of the bracket slot. Then, a pair of normal forces, which is called lingual root tipping moment, is generated. It is considered that greater the play between the archwire and the bracket, weaker the normal forces are, and thereby weaker the lingual root tipping moment transmitted to the incisor.

As mentioned above, the play is the most influential factor determining the type of anterior tooth movement in sliding mechanics combined with power arms. Thus, knowledge of the biomechanical principles of tooth movement in orthodontics cannot be directly applied in the actual clinical situation in which the play exists. Although one of the keys to an estimation of how the tooth will move is an appreciation of the relationship of a line of action of the retraction force and the CRe of a tooth, the effect of the archwire deflection within the bracket slot on force system acting on a tooth should also be taken into consideration (Figure 7).

By utilizing the power arm in sliding mechanics, the upward deformation and the torsion of the anterior segment of the archwire can be easily generated. The torsion of the archwire will produce a couple that works as an antitipping moment to the anterior tooth (Figure 7(b)). The couple causes lingual root tipping of the maxillary central incisor. The height of the retraction force on the power arm modifies the type of anterior tooth movement. Thus, in a simple way, the force system for the desired type of anterior tooth movement, such as controlled lingual crown tipping, bodily movement, or controlled lingual root tipping, could be provided by attaching various lengths of power arms onto an archwire. In other words, the employment of power arms prevents uncontrolled tipping or excessive lingual crown tipping during space closure. Nevertheless, quite long power arms are required to achieve controlled movement of anterior teeth when a play exists between the bracket and the archwire. In case, excessively long power arms are used, an impingement may be produced on the buccal mucosa. On the other hand, clinically appropriate length of the power arm allows any types of anterior tooth movement when a full-size archwire is used.

However, the situation in which there is no play between the archwire and the bracket is not clinical and has some disadvantages in sliding mechanics. That is, a friction will be produced that will prevent the tooth from sliding along an archwire. Therefore, the existence of archwire play in the bracket slot and its dimension should be taken into account in an actual clinical situation in order to precisely predict the tooth movement after going through the treatment. In addition, it is necessary to give full consideration to anatomic parameters that vary from one individual to another. Especially, an estimation of the CRe position is of utmost clinical importance prior to the treatment. It is because the length of the power arm itself does not have any significant importance. Since the height of the retraction force producing a certain type of tooth movement is closely related to the location of the CRe, an optimal power arm length should be back-calculated from the location of the CRe. To consider not only the relationship between the line of action of a retraction force and the location of the CRe of a tooth but also the effect of the archwire deformation, including the torsion within the bracket slot, will be a great help in establishing an optimal treatment plan and achieving speedy, effective, and accurate orthodontic tooth movement.

## Conclusions

1. A play between the archwire and the bracket has a great impact on the anterior tooth movement. Greater the play, more difficult it is to successfully produce a lingual root tipping on the incisor even with long power arms.
2. Not only the line of action of a force in relation to the CRe of a tooth but also a bracket/archwire play and the torsion of the archwire within the bracket slot should be taken into consideration to prescribe the power arm length, this will allow the desired tooth movement.
